# A Novel Approach to Characterizing Patterns of Real-World Upper-Extremity Use During Walking and Activities of Daily Living in People with Subacute Stroke

**DOI:** 10.3390/s26154881

**Published:** 2026-08-03

**Authors:** Aishwarya Shenoy, Kit B. Beyer, William McIlroy, Karen Van Ooteghem, Kyle S. Weber, Janice J. Eng, Jennifer Yao, Sean P. Dukelow, Gentson Leung, Michael D. Hill, Robert Teasell, Adria Quigley, Marilyn Mackay-Lyons, Anita Mountain, Nathania Liem, Benjamin R. Ritsma, Mark T. Bayley, Courtney L. Pollock

**Affiliations:** 1 Department of Physical Therapy, University of British Columbia (UBC), Vancouver, BC V6T 1Z3, Canada; aishshen@student.ubc.ca (A.S.);; 2 Rehabilitation Research Program, Centre for Aging SMART, Vancouver, BC V5Z 1M9, Canada; 3 Graduate Program in Rehabilitation Science, University of British Columbia (UBC), Vancouver, BC V6T 1Z3, Canada; 4 Department of Kinesiology and Health Sciences, University of Waterloo, Waterloo, ON N2L 3G1, Canada; 5 Ontario Brain Institute, Toronto, ON M5H 3W4, Canada; 6 Department of Clinical Neurosciences, Hotchkiss Brain Institute, University of Calgary, Calgary, AB T2N 4N1, Canada; 7 Lawson Research Institute at St. Joseph’s Health Care London, London, ON N6A 4V2, Canada; 8 Department of Physical Medicine and Rehabilitation, Schulich School of Medicine and Dentistry, Western University, London, ON N6A 5C1, Canada; 9 School of Physiotherapy, Dalhousie University, Halifax, NS B3H 4R2, Canada; 10 Department of Medicine, Physical Medicine and Rehabilitation, Dalhousie University, Halifax, NS B3H 4K4, Canada; 11 Department of Biomedical Sciences, University of Windsor, Windsor, ON N9B 3P4, Canada; 12 Department of Physical Medicine and Rehabilitation, Queen’s University, Providence Care, Kingston, ON K7L 4X3, Canada; 13 KITE Research Institute, University Health Network, Toronto, ON M5G 2A2, Canada; 14 Division of Physical Medicine and Rehabilitation, Department of Medicine, University of Toronto, Toronto, ON M5S 1A1, Canada

**Keywords:** stroke, wearable sensors, upper extremity, inertial measurement unit, remote monitoring, stroke rehabilitation

## Abstract

In accelerometer-based measurement, the inclusion of upper extremity (UE) activity during walking can overestimate real-world functional UE use in people post-stroke. This study introduces a comprehensive, accelerometer-based approach to examining real-world UE use, including the contribution of UE activity during walking, and compares UE use during walking versus ADL-based activities. People with subacute stroke wore bilateral wrist and ankle inertial measurement units (IMU) for one week (24 h/day). Agreement between UE-Total, UE-Continuous Walking and UE-ADL measures were assessed using Bland–Altman analyses. Variables were modeled using linear regression to explore step count, mobility aid use and UE motor impairment as predictors of IMU measures of real-world UE use. Agreement between UE-Total and UE-ADL measures showed minimal bias. Differences between UE-Continuous Walking and UE-ADL varied by mobility aid. UE-Continuous Walking was associated with increased UE activity and relatively greater paretic arm use compared with UE-ADL. Across UE-Continuous Walking and UE-ADL, step count was the strongest predictor of real-world UE use. UE activity during continuous walking exhibits different use patterns as compared to ADL-based activity. Accordingly, separating continuous walking-related UE activity from ADL-based UE use may improve clinical interpretation of IMU measures of real-world UE use in individuals with subacute stroke.

## 1. Introduction

Approximately 80% of people post-stroke have upper extremity (UE) impairments and 50% demonstrate challenges integrating their paretic arm into activities of daily living (ADL) [1,2]. Regaining the ability to use the paretic UE after stroke is a common goal for many people post-stroke, as it enables increased independence in productive and meaningful daily activities while improving overall quality of life [2]. Unfortunately, recovery of UE function does not occur at the same rate or to the same extent as the lower extremity [3,4,5], highlighting the distinct rehabilitation needs of the UE. Prior research has established the importance of incorporating the affected UE into ADLs to promote recovery [6]. For this reason, there has been a growing emphasis on measuring UE use in real-world conditions to obtain objective data on UE activity throughout the day. Such data have the potential to help clinicians tailor treatment plans that better support functional recovery and real-world arm use in people post-stroke. 

Aiming to better characterize real-world UE use, prior studies have used wearable sensors to obtain objective measures of daily UE activity, recognizing that clinical assessments may not capture real-world UE performance [7,8,9,10,11,12,13,14,15,16]. Results from these studies indicate that individuals with stroke exhibit greater asymmetry in UE use, relying more on the non-paretic arm and less on the paretic arm. This pronounced imbalance in UE use has prompted ongoing efforts to better understand real-world UE use patterns after stroke and to develop measures that more accurately capture functional arm activity. In this context, previous studies have suggested that including UE activity during walking can overestimate the amount of purposeful use of the UE in real-world conditions in people post-stroke [8,16,17,18]. However, these studies did not differentiate continuous walking, where rhythmic arm swing is more likely to occur as an automatic gait-related movement [19], from intermittent stepping, which is more likely to occur during ADLs. This suggests that active use of the UEs in real-world conditions should be examined in three complementary ways: (1) UE-Total Activity, which reflects both purposeful and non-purposeful movements; (2) UE-ADL, capturing arm movements that likely reflect functional use during everyday activities (e.g., self-care and occupational tasks), while still accounting for intermittent steps taken during ADLs, and (3) UE-Continuous Walking, capturing rhythmic arm swing during continuous bouts of walking. Distinguishing UE activity across these three contexts allows clinicians and researchers to look at the functional integration of the affected arm into daily tasks, which can have implications for stroke recovery and rehabilitation planning.

Increased use of the paretic arm is associated with less severe motor impairment [20,21]. However, improvements in clinical measures of UE functional recovery do not consistently translate to increased daily use [4,8,9,10,22]. Additionally, walking ability (e.g., speed, independence) [23] and volume [24] have been reported to be associated with daily UE use, suggesting walking should be considered when evaluating real-world UE activity. It should also be noted that use of a mobility aid not only impacts participation in complex and social activities [25] but also UE use patterns during walking [9], as the engagement of both arms differs across aids (e.g., swinging the non-paretic arm with a cane versus a fixed position of both arms with a wheeled walker). Taken together, this highlights the complexity of factors that should be considered when assessing real-world arm use during gait and non-gait related activities.

One of the main limitations of studies that have addressed the effect of walking on real-world UE use is the reliance on ‘activity counts’ for quantifying UE activity [15,23]. Activity counts quantify acceleration within a window of time (i.e., an epoch) using raw accelerometer data and have been reported to be the most commonly used metric for assessing real-world UE activity [10,13,26,27]. However, the algorithms used to derive them vary across devices, limiting standardization and reproducibility across studies [10,13,28]. Furthermore, although the subacute period of stroke recovery (<6 months post-stroke) is characterized as a critical window during which substantial gains in functional ability are observed [29,30], very few studies have examined the contribution of arm swing activity during walking on daily, real-world UE use in subacute stroke [9,18]. While Leuenberger et al. [18] included subacute participants, their sample size was small (n = 7) with data collapsed between the subacute and chronic (>6 months post-stroke) periods. Bezuidenhout et al. [9] used activity counts for gait detection for the purpose of exploring differences in UE symmetry during ambulatory and non-ambulatory activities. This approach may have introduced inaccuracies in identifying ambulation, as slower speeds of walking, commonly observed in people post-stroke, may not meet the threshold for step detection [31]. Consequently, the current recommendation for accurate accelerometer-based gait detection in individuals with slower gait speeds is to place sensors on the lower limb, as this provides more precise measurements of gait cycle kinematics (e.g., heel strike, toe-off) [32,33]. The present study addresses these methodological limitations to provide a better understanding of UE use patterns in real-world conditions among individuals with subacute stroke.

The overarching aim of this study was to explore the contribution of UE activity measured during continuous walking on overall measures of UE use in real-world conditions in people with subacute stroke. This was addressed by examining: (1) how the measurement of UE activity during continuous walking contributes to total UE activity, (2) differences in UE activity during continuous walking and UE activity during ADL-based activities and how these differences vary by mobility aid use, and (3) whether daily step count, mobility aid use, and UE motor impairment (Fugl Meyer Assessment-UE) predict UE activity during continuous walking and ADL-based activities. It was hypothesized that: (1) the inclusion of UE activity occurring during continuous walking will inflate various metrics of overall daily UE use, (2) UE activity would be more symmetrical during continuous walking than during ADL-based activities and that cane users would show greater UE asymmetry during continuous walking compared to individuals without a mobility aid, and (3) daily step count, use of mobility aid, and level of UE motor impairment would influence UE use in both contexts (e.g., ADL-based activities and continuous walking).

## 2. Materials and Methods

### 2.1. Study Design and Participants

Data are from the Canadian Maraviroc Randomized Controlled Trial to Augment Rehabilitation Outcomes After Stroke (CAMAROS) (NCT04789616) and the Stroke Active Transition Home Program (NCT06119230). CAMAROS is designed to examine the effect of Maraviroc combined with exercise to improve upper and lower extremity recovery post-stroke. Participants in this trial had wearable sensor collections at: baseline (admission to trial), 4 and 8 weeks baseline and 6 months post-stroke. The Stroke Active Transition Home Program was designed to explore the feasibility of using wearable sensors to remotely monitor activity levels in individuals transitioning from structured rehabilitation to home. Participants in this trial had wearable sensor collections at: baseline (admission to trial), 3 weeks, 7 weeks and 3 months post baseline. Inclusion criteria for the CAMAROS study included: <8 weeks post-stroke, presenting with anterior circulation ischemic stroke, and upper and/or lower extremity impairment requiring inpatient rehabilitation. Inclusion criteria for the Stroke Active Transition Home Program included: ability to walk 10 m with or without a mobility aid and ability of the participant or a family member to speak English. This analysis included data from trial participants who were admitted into inpatient rehabilitation facilities across Canada between July 2022 and December 2025. Each original study received ethical approval from the Research Ethics Boards at the respective coordinating institutions and participating sites. All participants provided written informed consent prior to participation.

All participants began participation during the subacute stage of recovery. To enable pooled analyses, data collection time points were matched across studies to ensure all participants were assessed at comparable time points. This resulted in using the week 8 collection for CAMAROS participants and baseline for Stroke Active Transition Home participants, which was approximately 14.4 weeks post-stroke for both groups. Participants were excluded from the analysis if they used a wheelchair for daily mobility. At the time of collection, participants in the Stroke Active Transition Home Program were participating in an outpatient rehabilitation program receiving multi-disciplinary therapy (e.g., physical therapy, occupational therapy, and/or speech therapy) for three to five days a week. Participants in CAMAROS were either in inpatient rehabilitation or at home.

### 2.2. Data Collection

#### 2.2.1. Demographic and Clinical Characteristics

Descriptive measures of participants included age, time since stroke, type of stroke, mobility aids used, paretic side and dominant side. With respect to clinical measures, the 6 Minute Walk Test (6MWT) and Fugl Meyer Assessment (FMA) for upper and lower extremities were collected to assess the severity of impairment post-stroke [34]. FMA-UE scores between 0 and 29 indicate severe impairment, 30 and 45 indicate moderate impairment, and above 45, mild impairment [35].

#### 2.2.2. Wearables (IMU) Data Collection

The methodology for remote collection of wearable sensor data was the same in both the CAMAROS and Stroke Active Transition Home program studies. Participants wore bilateral wrist and ankle Axivity AX6 (Newcastle, UK) IMUs for seven days (24 h/day), only removing them when showering or entering a swimming pool. Devices were inserted into custom-made Velcro straps (Fabrifoam, Exton, PA, USA) with labels indicating appropriate location (e.g., right wrist, left wrist). Wrist sensors were placed on the dorsal side of the wrist consistent with previous studies investigating real-world upper extremity activity in individuals with stroke [8,14,18,36]. Ankle sensors were placed on the shank proximal to the lateral malleolus, similar to prior studies investigating real-world gait in people with stroke [37,38]. Participants were provided with instructions on how to don and doff the sensors to avoid incorrect wear of the sensors. IMU data was sampled at 50 Hz with an accelerometer range of ±16g and gyroscope range of 2000 deg/s. All data were stored using onboard storage. At the end of collection, the data were downloaded by a member of the research team and transferred to a secure server. All four devices were synchronized at both the beginning and end of the collection period to account for potential clock drift over multiple days of wear. Synchronization involved 5 to 10 device rotations with 5 to 10 s of rest between rotations, allowing for an easily detectable repeating square waveform in the accelerometer.

### 2.3. Data Analysis

#### 2.3.1. Data Processing

Sensor data for participants were included if they wore all four sensors for at least two days (>8 waking hours per day), termed a ‘valid day’. These criteria have been used in the past to monitor UE activity in real-world conditions for people post-stroke [39]. The raw wrist sensor data were filtered with a 5 Hz low-pass, 4th-order Butterworth filter. Vector magnitude (VM) was calculated using the filtered accelerometer data to obtain a combined acceleration measure across all three axes (vertical, horizontal and perpendicular) with gravity removed (1).

(1) $$VM=\sqrt{a_{x}^{2}+a_{y}^{2}+a_{z}^{2}}-1$$

#### 2.3.2. Epoch Selection

Prior studies monitoring UE activity during free-living in people post-stroke have used 1-s epochs to calculate activity counts [8,14,27]. Similar to Bezuidenhout et al. (2021) [32], a 5-s epoch was used due to its ability to avoid fragmenting movements and provide a stable representation of functional activity. The 5-s epochs were used to calculate the average vector magnitude (AVM) (2), which was obtained by averaging all VM values within each epoch and then converting the result into acceleration values (milli-g).

(2) $$AVM\;(milli\;g)=\frac{1}{N}\sum_{i=5}^{N}{VM}_{i}\times 1000$$

#### 2.3.3. Data Processing Using Nimbalwear Pipeline

NiMBaLWear is an open source, Python based, modular wearable sensor data processing pipeline that quantifies multiple health-related domains, including physical activity, mobility (steps), sleep, and sedentary behavior [40]. It has previously been used to process IMU data from individuals with neurodegenerative and cerebrovascular post-stroke [40,41].

Wrist accelerometer data were processed using the pipeline to identify periods of non-wear and sleep using the algorithms detailed in Beyer et al. [40]. These periods were excluded so that analyses focused on when participants were awake and wearing the device. Gait bouts were also identified using the pipeline’s gait analytics module using ankle gyroscope data. A gait bout was defined as a minimum of two steps, with each step occurring within 2 s of the previous step [42]. Steps that occurred within gait bouts were classified as steps taken during Continuous Walking (“UE-Continuous Walking”), whereas steps that did not meet the bout criteria were classified as “UE-ADL” and considered potentially associated with activities of daily living (e.g., meal preparation, self-care activities).

### 2.4. Primary IMU Measures of UE Activity

AVM values were used to calculate two primary IMU Measures of arm activity: bilateral magnitude and magnitude ratio. These measures were selected a priori because they can capture complementary dimensions of real-world UE use. Bilateral magnitude quantifies the acceleration magnitude of activity across both arms by summing the paretic and non-paretic AVM values (3) [43]. A value of 0 indicates that no movement occurred in either UE and increasing bilateral magnitude values would reflect increasing magnitude of activity across one limb or both limbs. Magnitude ratio reflects the relative contribution of each UE by expressing the log-transformed ratio of paretic to non-paretic AVM, providing an estimate of interlimb symmetry (Equation 4) [43]. Values closer to zero indicate more symmetric use, whereas positive and negative values indicate greater dominance of the paretic and non-paretic limb, respectively. In instances of unilateral activity, a ratio of −7 is assigned when only the non-paretic UE was being used and +7 when only the paretic UE was being used.

(3) $$Bilateral\;Magnitude={AVM}_{paretic}+{AVM}_{non-paretic}$$

(4) $$Magnitude\;Ratio=\log\frac{{AVM}_{paretic}+1}{{AVM}_{non-paretic}+1}$$

While the nimbalwear pipeline reduced mean calibration error to 3.8 mg after autocalibration [40], epochs where no movement occurred or where movement was likely due to this residual calibration error (bilateral magnitude ≤ 7.6) [40] were removed to ensure that only active movement periods were included in the analysis of bilateral magnitude and magnitude ratio.

### 2.5. Secondary IMU Measures of UE Activity

Duration of use is a commonly used measure to report UE activity in individuals with stroke [10,13]. Thus, the following secondary variables were calculated: duration of (1) concurrent arm use (2) unilateral paretic and (3) unilateral non-paretic UE use. Epochs with wrist AVM values below 3.8 mg were removed to account for calibration error. Concurrent use was defined as any epoch during which both arms were above the movement threshold (3.8 mg). Due to the use of 5-s epochs, concurrent use may not accurately represent true bilateral use (simultaneous use of both arms). However, it does reflect instances in which both arms are involved in completing a task, such as opening a cupboard with the paretic UE and retrieving an item with the non-paretic UE. Unilateral arm use was defined as epochs in which only one arm exceeded the threshold. Epochs in which only the paretic arm exceeded the threshold were classified as “unilateral paretic”, whereas epochs in which only the non-paretic arm exceeded the threshold were classified as “unilateral non-paretic”. Epochs where no movement occurred across both limbs were classified as “sedentary”. Tallies of each category of epoch were then converted into hours of use.

### 2.6. Data Preparation

Arm activity was then separated into three categories: (1) total UE activity—all use of the UE throughout the collection period (UE-Total), (2) ADL-based UE activity (UE-ADL), and (3) UE activity during continuous walking (UE-Continuous Walking).

### 2.7. Statistical Analysis

Descriptive statistics of demographic, clinical and sensor-derived activity characteristics were calculated. For measures without a normal distribution, the median and interquartile range were reported. For sensor-derived activity characteristics, daily values were summarized for all valid days within each participant, and participant-level summaries were used to calculate group-level descriptive statistics. Participants using a single point cane, quad cane, or singular walking pole were grouped under the “Cane” category. The level of significance was set to 5%. 

For objective 1, Bland–Altman plots were used to visualize the agreement between UE-Total (i.e., ADL-based activities and continuous walking) and UE-ADL (i.e., activity during purposeful UE activities) separately for bilateral magnitude and magnitude ratio to quantify the contribution of UE-Continuous Walking to UE-Total. For objective 2, Bland–Altman plots were used to visualize the agreement between UE-ADL and UE-Continuous Walking (i.e., UE activity due to just walking) separately for bilateral magnitude and magnitude ratio to evaluate whether UE activity patterns during continuous walking differ from those observed during ADL-based activities. For all Bland–Altman plots, points were color-coded by volume of walking (average daily step count), level of UE impairment (FMA-UE), and type of mobility aids used to visualize if any patterns emerged. Steps were color-coded into 3 groups: (1) 0< step count < 3000, (2) 3000 ≤ step count < 6000 and (3) step count ≥ 6000. Level of impairment was color-coded into 3 groups: (1) Mild, (2) Moderate and (3) Severe. Lastly, mobility aid use was also color-coded into 3 groups: (1) No Gait Aid, (2) Cane and (3) Wheeled Walker.

Mean difference (bias) and limits of agreement (LoA) were reported for all Bland–Altman plots. A proportional bias analysis was run for plots indicating that the magnitude of bias was associated with the mean of the measure. The differences between the two measures were regressed on the mean of measures. A significant slope indicated the presence of proportional bias.

For objective 3, multiple linear regression models were estimated using ordinary least squares (OLS) in Python (version 3.12) with the statsmodel package [44] to examine predictors of UE activity during ADL-based activities and continuous walking. Participants using a cane or wheeled walker were collapsed into a “Gait Aid” group due to small sample sizes in each category. Linear regression models were conducted on five different dependent variables: bilateral magnitude, magnitude ratio, concurrent arm use time, unilateral paretic and unilateral non-paretic use time. Each dependent variable was explored for explaining the variance (R^2^) in UE activity during ADL-based activities and continuous walking. Across all models, the predictors included step count (centered per 1000 steps), mobility aid use (binary), and FMA-UE score (centered per 5-point increase). Predictors were centered to aid with the interpretation of the regression coefficients. Step count variables were defined according to the model being tested. Step counts from ADL-based activities were used for UE-ADL models, and step counts from continuous walking were used for UE-Continuous Walking. Variables (e.g., daily step count, FMA-UE and mobility aid use) that demonstrated visual patterns from the Bland–Altman in objective 1 and 2 determined the order of predictors that were entered hierarchically in the two models. Furthermore, previous work has shown that FMA-UE may be a weak predictor of real-world UE use [14]. Accordingly, Model 1 included step count and mobility aid use to account for basic mobility factors that may influence UE activity. Model 2 added FMA-UE to examine whether UE motor impairment contributed additional explanatory power beyond Model 1 predictors. The incremental change in variance explained (ΔR^2^) was calculated for each block to quantify their relative contributions to the outcome. Although predictors were entered hierarchically in two blocks, only the results from the full model (Model 2) are reported.

## 3. Results

### 3.1. Participants

Demographic and clinical characteristics for the two studies pooled together are presented in Table 1. Forty-six participants (28 males and 18 females) with subacute stroke (14.4 weeks ± 4.9 weeks post-stroke) were included in the analysis (Table 1). The majority of participants had experienced an ischemic stroke (n = 37), with the remainder experiencing an intracerebral or subarachnoid hemorrhagic stroke (n = 9). At the time of collection, 14 participants were active in inpatient rehabilitation and 32 participants were living at home; 18 of the participants living at home were participating in a structured outpatient rehabilitation day program. Post-stroke impairment as measured by the FMA for both upper and lower extremities ranged from mild to severe (FMA-UE:8 to 66; FMA-LE: 13 to 34). Participants varied in their use of mobility aids: no gait aid (n = 21), cane (n = 6), quad cane (n = 2), walking pole (n = 5), and wheeled walker (n = 12). Walking distance on the 6MWT ranged from 34 m to 598 m, highlighting the range of walking ability among this subacute sample. 

Since participants were pooled from two different studies, participant and clinical characteristics were also examined by study (Table A1). No differences were observed for time since stroke and motor impairment as shown by the FM-LE and FM-UE. Participants in CAMAROS were older and demonstrated lower mobility performance (6-min walk test) and activity levels (average step count) than participants in the Post-Stroke Active Transition Home Program. These differences were addressed analytically by including step count and gait aid use as predictors in the OLS models.

Activity characteristics as measured by the IMUs are reported in Table 2. Participants wore the sensors for 6.3 days ± 1.5 days and 11.9 ± 2.5 waking hours per day. Daily step counts from continuous walking had a median of 3740 steps (IQR: 2065 to 7739). Participants showed asymmetry (median magnitude ratio: −0.9, IQR: −1.3 to −0.5) for UE-ADL, with a slightly higher contribution of the non-paretic arm. For UE-Continuous Walking, participants were more symmetric (median magnitude ratio: −0.2, IQR: −0.5 to 0.1) than in UE-ADL. Across all participants, the bilateral magnitude for UE-Continuous Walking (median: 59.1, IQR: 40.6 to 88.1) was higher compared to UE-ADL (median: 25.7, IQR: 21.7 to 29.4). Table 3 provides hours of UE use for UE-Total and UE-ADL by type of mobility aid. For UE-Total, participants not using a gait aid showed higher concurrent and unilateral paretic arm use time compared to cane and wheeled walker users. Cane users showed the highest unilateral non-paretic arm use time compared to no gait aid and wheeled walker users.

### 3.2. Bland–Altman Analysis

#### 3.2.1. Objective 1: Contribution of UE-Continuous Walking to UE-Total

**Figure 1:**
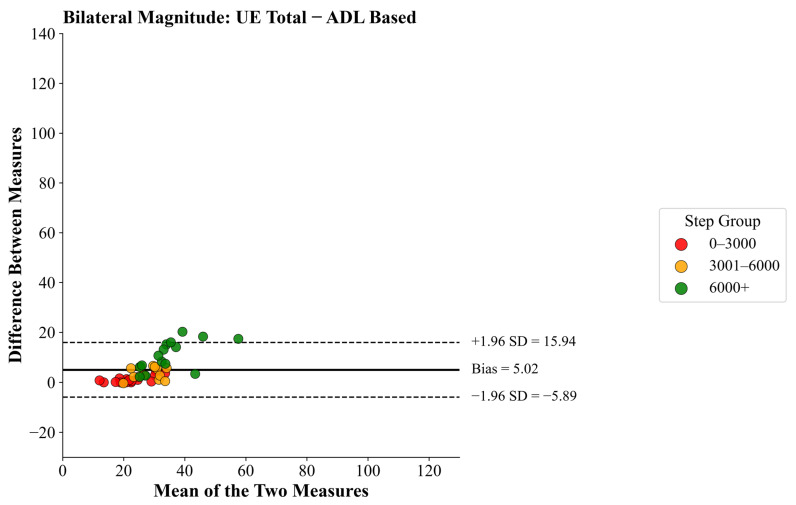
Bland–Altman showing agreement between UE-Continuous Walking and UE-Total for bilateral magnitude. Each point represents a participant, color-coded by average daily step count (0–3000 = red, 3000–6000 = orange, 6000+ = green).

**Figure A1:**
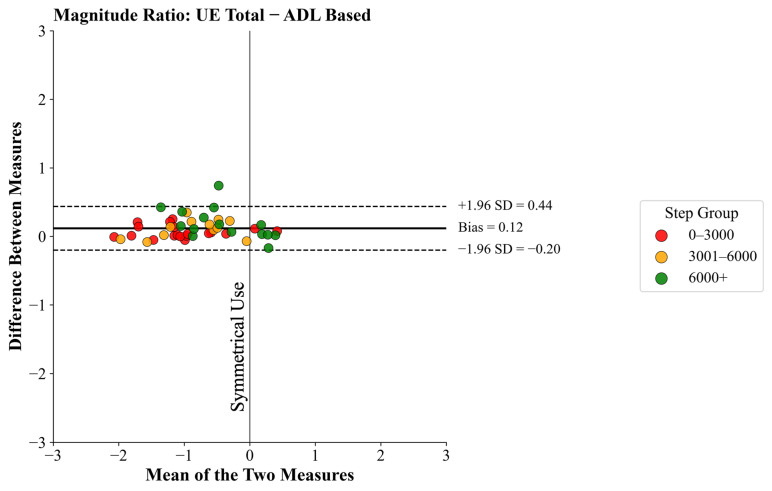
Bland–Altman showing agreement between UE-Continuous Walking to UE-Total for magnitude ratio. Each point represents a participant, color-coded by average daily step count (0–3000 = red, 3000–6000 = orange, 6000+ = green).

Across all participants, bilateral magnitude had a mean bias of 5.024 (95% LOA: −5.890 to 15.938; Figure 1) when looking at the difference between UE-Total and UE-ADL, indicating that inclusion of UE-Continuous Walking slightly overestimates purposeful arm activity. Linear regression indicated a significant positive proportional bias (slope = 0.490, 95% CI [0.355, 0.625]; *p* < 0.001; R^2^ = 0.550), indicating that as overall arm activity increases, the discrepancy between measures also increases. This suggests that at higher levels of total arm activity, walking-related movements contribute disproportionately more to arm activity. This interpretation is consistent with the observational findings, where individuals with higher step counts exhibited larger differences (Figure 1). For magnitude ratio, the mean bias was 0.121 (95% LOA: −0.195 to 0.437; Appendix A Figure A1), indicating that UE-Total reflects a shift toward greater paretic side use relative to UE-ADL activity, suggesting that UE-Continuous Walking may involve comparatively greater paretic contribution than UE-ADL. For magnitude ratio, there was no evidence of proportional bias (slope = −0.017, 95% CI: [−0.087, 0.053]; *p* = 0.624; R^2^ = 0.005).

#### 3.2.2. Objective 2: Differences in UE Use Between UE-Continuous Walking and UE-ADL

**Figure 2:**
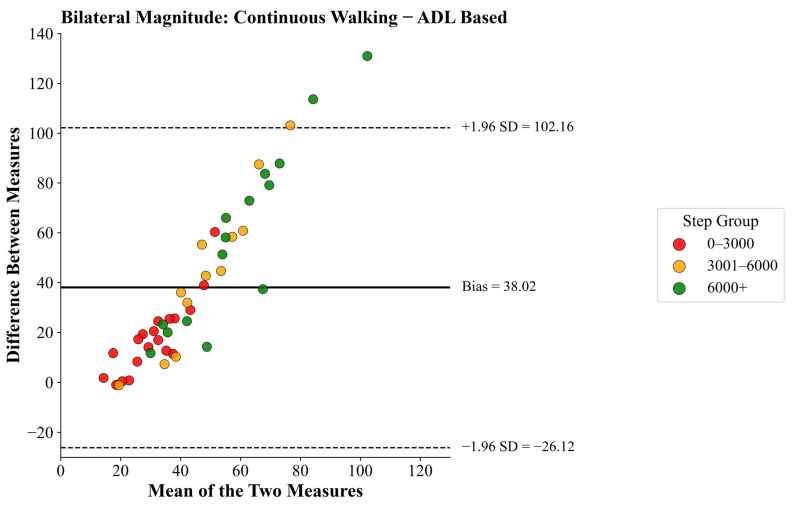
Bland–Altman plot looking at the agreement between UE-Continuous Walking and UE-ADL for bilateral magnitude.

**Figure A2:**
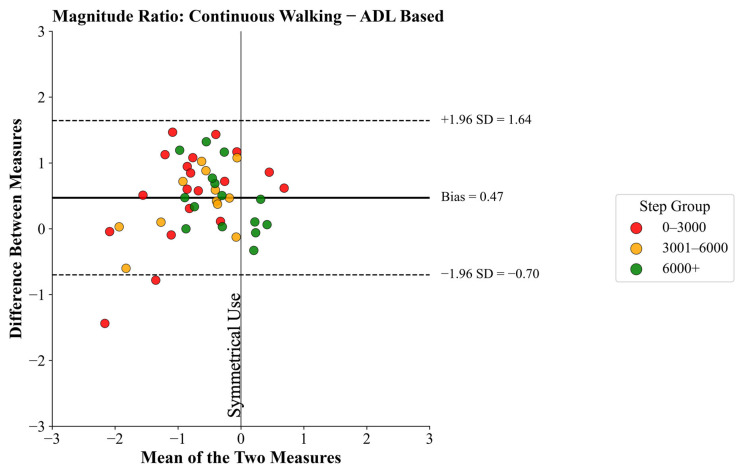
Bland–Altman showing agreement between UE-Continuous Walking and UE-ADL for magnitude ratio. No clear visual pattern between average daily step count and agreement between UE-Continuous Walking and UE-ADL.

Bilateral magnitude had a mean bias of 38.018 (95% LOA: −26.121 to 102.158; Figure 2) when looking at the difference between UE-Continuous Walking and UE-ADL, suggesting that arm movement might be higher in acceleration magnitude during continuous walking compared to ADL-based activities. A significant positive proportional bias was observed (slope = 1.602, 95% CI [1.432, 1.773]; *p* < 0.001, R^2^ = 0.891) when looking at the differences between the UE-Continuous Walking and UE-ADL, indicating that at higher levels of UE use, UE-Continuous Walking demonstrates increasingly greater values than UE-ADL. For magnitude ratio, the mean bias was 0.463 (95% LOA: −0.729 to 1.654; Appendix A Figure A2), consistent with findings from Objective 1, indicating a directional shift toward greater paretic side use during UE-Continuous Walking compared with UE-ADL activity. No proportional bias was observed (slope = 0.145, 95% CI: [−0.120, 0.409]; *p* = 0.276, R^2^ = 0.027), suggesting that this difference was consistent across all levels of UE symmetry. When looking at agreement between UE-Continuous Walking and UE-ADL, differences for bilateral magnitude and magnitude ratio showed greater dispersion, this was reflected in the wide LOA for both measures, suggesting interindividual variability in UE use and mobility.

**Figure 3:**
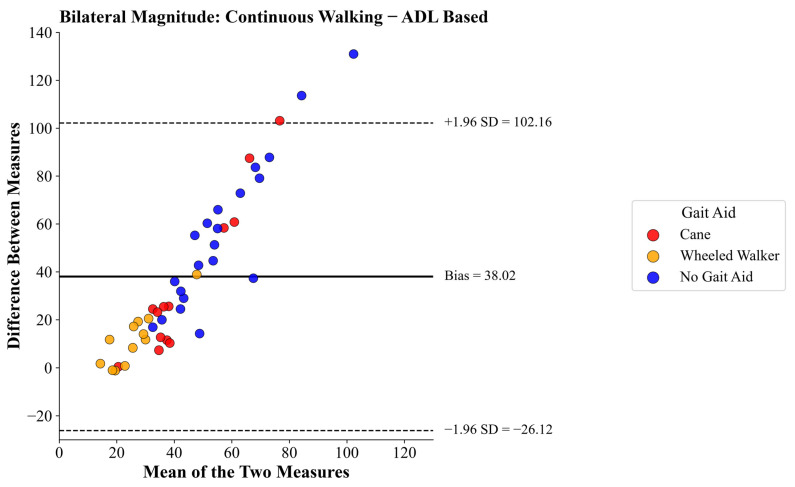
Bland–Altman plot comparing UE-Continuous Walking and UE-ADL for bilateral magnitude. Each point represents a participant, color-coded by the type of mobility aid used. Cane users are distributed throughout the plots, making patterns difficult to interpret. No gait aid users have larger means and differences compared to wheeled walker users, suggesting that type of mobility aid used may influence bilateral magnitude.

**Figure A3:**
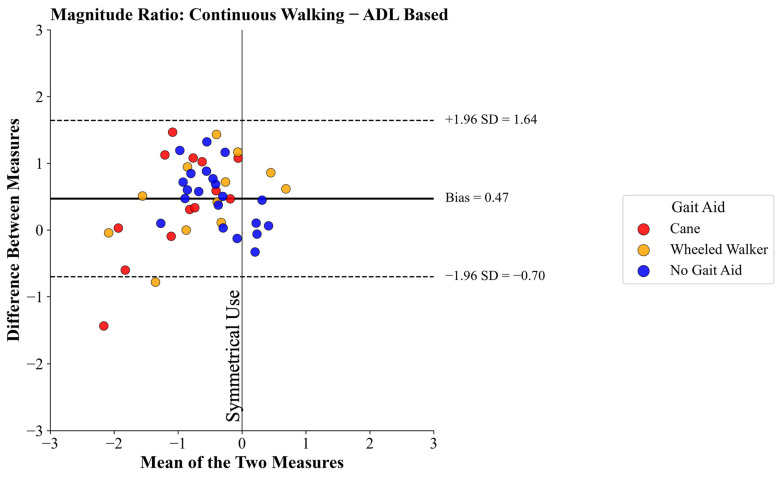
Bland–Altman plots visualizing the agreement between UE-Continuous Walking and UE-ADL for magnitude ratio, with each participant’s point color-coded by type of mobility aid use.

**Figure A4:**
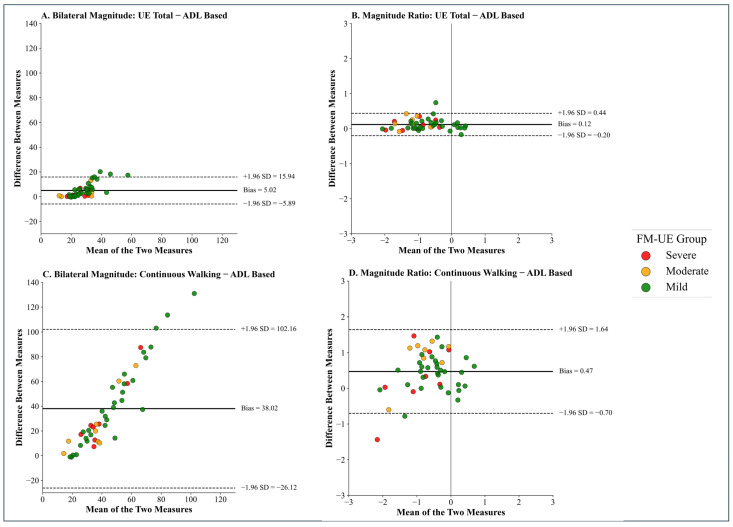
Bland–Altman plots to visualize agreement between UE-Total and UE-ADL for bilateral magnitude (**A**) and magnitude ratio (**B**). Second row of Bland–Altman plots visualize the agreement between UE-Continuous Walking and UE-ADL for bilateral magnitude (**C**) and magnitude ratio (**D**). Each point represents a participant, color-coded by their level of UE impairment as characterized by the FMA-UE.

When looking at the agreement between UE-Continuous Walking and UE-ADL, various observational patterns emerged for bilateral magnitude when the points in the Bland–Altman plots were color-coded by step count and mobility aid use. For step count, participants with higher step counts (6000+ steps) generally showed higher bilateral magnitude and larger differences, whereas those with lower step counts (0–3000 steps) had smaller bilateral magnitude values and differences (Figure 2). For mobility aids, those using wheeled walkers had lower bilateral magnitude values and differences compared to those using a cane or no gait aid (Figure 3). Step count and mobility aid use did not show any patterns for magnitude ratio, suggesting that these variables may not influence symmetry of UE use (Appendix A Figure A2 and Figure A3). Lastly, UE motor impairment (FMA-UE) (Appendix A Figure A4) did not show any clear pattern in relation to either bilateral magnitude or magnitude ratio. Together, these observations suggest that step count and mobility aid use may influence bilateral magnitude, but not the magnitude ratio. 

#### 3.2.3. Objective 2: Differences in UE Use Between UE-Continuous Walking and UE-ADL by Mobility Aid

**Figure A5:**
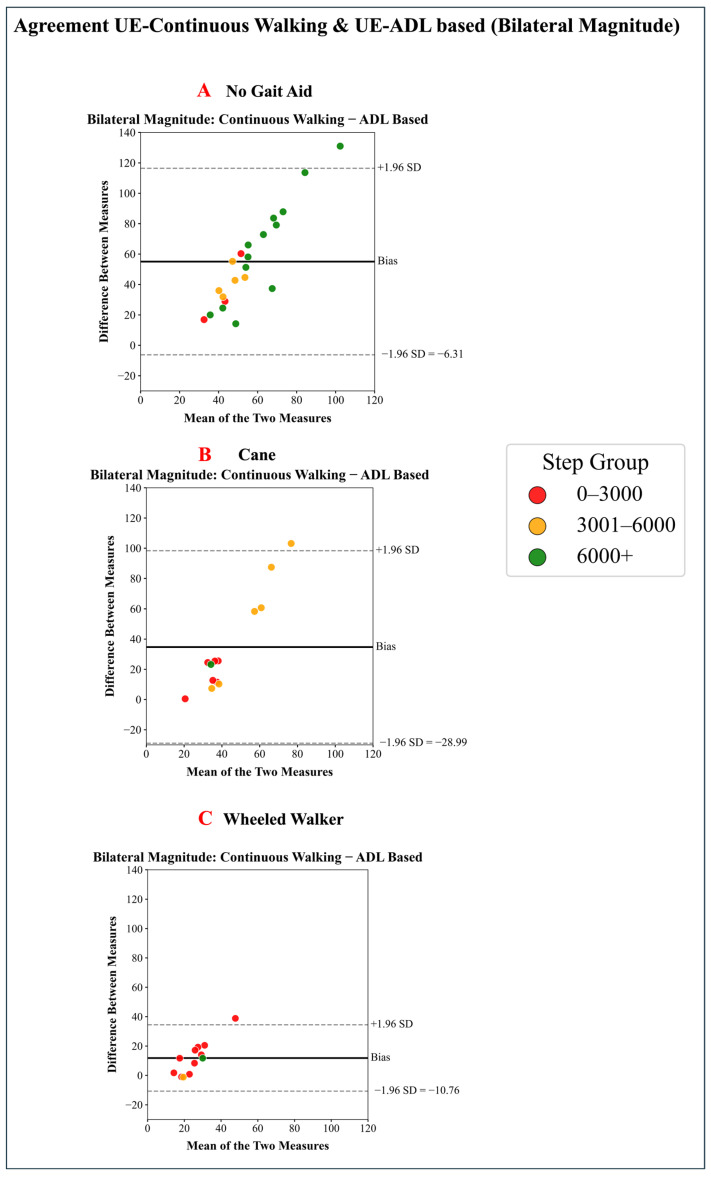
Subgroup analyses of differences between UE-Continuous Walking and UE-ADL for bilateral magnitude, by mobility aid type. Participants without gait aids showed the highest bilateral magnitude (**A**), followed by cane users (**B**) and wheeled walker users (**C**). Higher volume of walking (average daily step count) continued to show a pattern with higher mean bilateral magnitude.

Based on the observational trends seen in Figure 2, subgroup analysis was conducted to explore the agreement between UE-Continuous Walking and UE-ADL within each mobility aid group. There were 21 participants who used no gait aid, 13 who used a cane and 12 who used a wheeled walker. Bland–Altman plots revealed that the bias for bilateral magnitude was higher in participants using no gait aid (n = 21; 55.055; 95% LOA −6.307 to 116.417; Appendix A Figure A5A) compared to cane (n = 13; 34.663; 95% LOA −28.993 to 98.320; Appendix A Figure A5B) and wheeled walker users (n = 12; 11.840; 95% LOA −10.763 to 34.443; Appendix A Figure A5C), suggesting that mobility aid use impacts the magnitude of arm movement acceleration during continuous walking. For magnitude ratio, all mobility aid groups showed a positive bias, indicating that magnitude ratio was higher during UE-Continuous Walking compared to UE-ADL. This suggests an increased relative contribution of the paretic arm during walking compared to ADL-based activities. 

**Figure 4:**
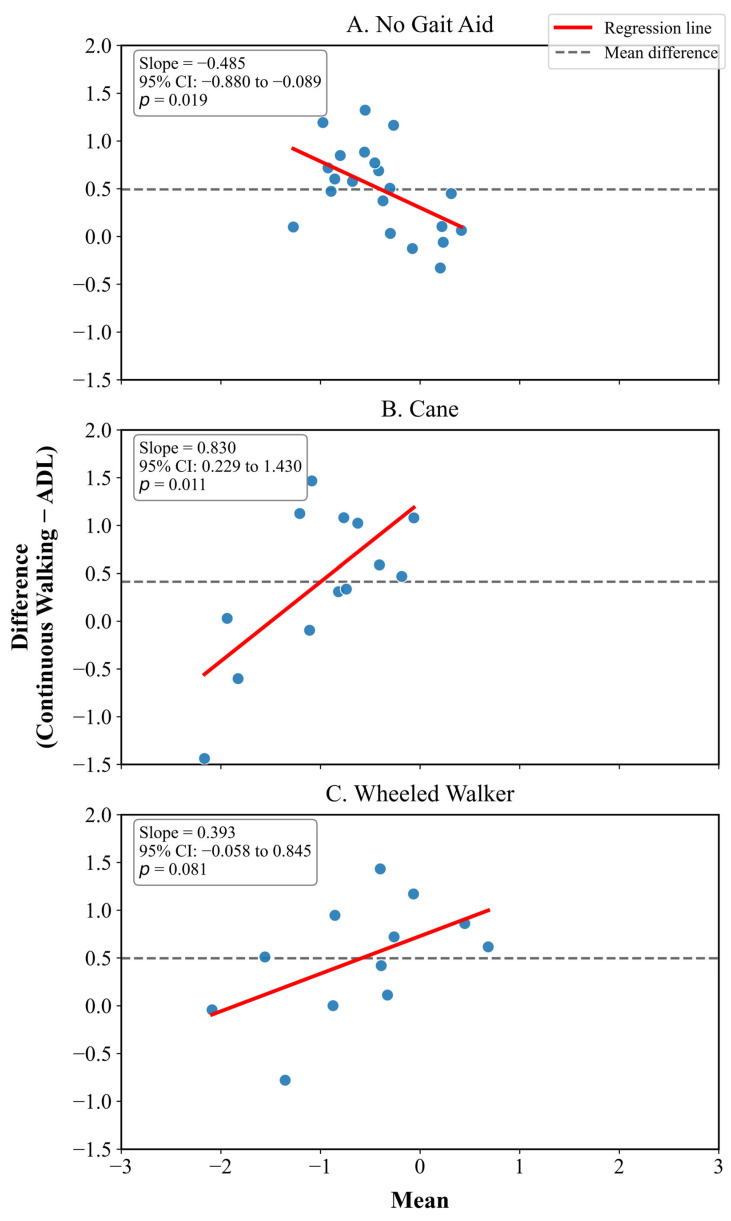
Bland–Altman plots illustrating proportional bias in magnitude ratio between UE-Continuous Walking and UE-ADL grouped by mobility aid type. *Y*-axis is the difference in magnitude ratio for UE-Continuous Walking and UE-ADL (**A**) Participants without a gait aid showed proportional bias with a significant negative slope. (**B**) Cane users showed proportional bias with a significant positive slope. (**C**) For wheeled walker users, although there was a slightly positive slope, it was not statistically significant.

Magnitude ratio showed different patterns across the mobility aid groups (Figure 4). Proportional bias analysis for magnitude ratio plots showed a negative slope for individuals not using a gait aid (slope = −0.485, 95% CI: [−0.876, −0.086]; *p* = 0.019, R^2^ = 0.257); the differences were largest in individuals with higher asymmetry in UE use and minimal in those who were already symmetric (mean magnitude ratio = 0) (Figure 4A). In contrast, cane users had a positive slope (slope = 0.830, 95% CI: [0.229, 1.430]; *p* = 0.011, R^2^ = 0.457); participants who showed overall non-paretic dominance (mean < 0) showed negative differences and those who were closer to symmetrical use (mean = 0) showed positive differences. No proportional bias was observed for wheeled walker users (slope = 0.393, 95% CI: [−0.058, 0.845]; *p* = 0.081, R^2^ = 0.273), indicating that the difference in magnitude ratio between UE-Continuous Walking and UE-ADL did not depend on participants’ overall symmetry. These findings suggest that gait aid use may influence the symmetry of UE use during continuous walking.

### 3.3. Linear Regressions for Predictors of UE Activity

#### 3.3.1. Objective 3: Predictors of UE-ADL

Table 4 provides the linear regression results for the IMU measures of UE-ADL.

The regression model predicting bilateral magnitude was statistically significant (F(3,42) = 6.977, *p* < 0.001) and explained 28.5% of the variance in bilateral magnitude after adjustment for model complexity (Adjusted R^2^ = 0.285, R^2^ = 0.333, n = 46). For this model, step count was a significant predictor, with each additional 1000 steps associated with a 4.840-unit increase in bilateral magnitude (B = 4.840 per 1000 steps, 95% CI [1.830, 7.850], *p* = 0.002). Gait aid use showed a trend (B = −3.843, 95% CI [−7.795, 0.108], *p* = 0.056), suggesting that a participant’s use of gait aids may be a predictor of a lower bilateral magnitude. FMA-UE (B = 0.454, 95% [−0.146, 1.055], *p* = 0.134) was not a significant predictor of bilateral magnitude.

The regression model predicting magnitude ratio was statistically significant (F(3, 42) = 3.484, *p* = 0.024) and explained 14.2% of the variance in the outcome after adjustment for model complexity (Adjusted R^2^ = 0.142, R^2^ = 0.199, n = 46). FMA-UE was a significant predictor with a small effect size (B = 0.073, 95% CI: [0.009, 0.136], *p* = 0.026); each 5-point increase in FMA-UE was associated with a 0.073-point increase in magnitude ratio, suggesting increased incorporation of the paretic arm with less impairment. Step count (B = 0.021 per 1000 steps, 95% CI: [−0.298, 0.340], *p* = 0.893) and gait aid use (B = −0.127, 95% CI: [−0.546, 0.292], *p* = 0.544) were not significant predictors.

The regression model predicting unilateral paretic arm use time was statistically significant F(3, 42) = 3.866, *p* = 0.016, explaining 16% of the variance in the outcome after adjusting for model complexity (Adjusted R^2^ = 0.160, R^2^ = 0.216, n = 46). Step count was a significant predictor (B = 0.357, 95% CI: [0.062, 0.653], *p* = 0.019), as each additional 1000 steps was associated with a 0.357-h increase in paretic arm use. FMA-UE was also a significant predictor, with a small effect size (B = 0.071 95% CI: [0.012, 0.130], *p* = 0.020). A 5-point increase in FMA-UE was associated with a 0.071-h increase in paretic arm use. Gait aid use (B = −0.026, 95% CI: [−0.414, 0.362], *p* = 0.893) was not a significant predictor. 

The regression models for concurrent arm use time (F(3,42) = 2.393, *p* = 0.082) and unilateral non-paretic arm use time (F(3,42) = 2.414, *p* = 0.080) were not statistically significant. None of the predictors significantly explained the variance in these outcomes (all *p* > 0.05).

#### 3.3.2. Objective 3: Predictors of UE-Continuous Walking

Table 5 provides the linear regression results for the IMU measures of UE-Continuous Walking.

The regression model predicting bilateral magnitude was statistically significant (F(3, 42) = 9.255, *p* < 0.001); and explained 35.5% of the variance in the outcome after adjustment for model complexity (Adjusted R^2^ = 0.355, R^2^ = 0.398, n = 46). For this model, step count was a significant predictor, with each additional 1000 steps showing a 4.329-point increase in bilateral magnitude (B = 4.329 per 1000 steps, 95% CI [1.337, 7.320], *p* = 0.006). Gait aid (B = −16.483, 95% CI [−40.486, 7.521], *p* = 0.173) and FMA-UE (B = −0.635, 95% CI [−3.541, 2.271], *p* = 0.661) were not significant predictors of bilateral magnitude.

The regression model predicting concurrent arm use time was statistically significant (F(3, 42) = 104.2, *p* < 0.001) and explained 87.3% of the variance after adjusting for model complexity (Adjusted R^2^ =0.873, R^2^ = 0.882, n = 46). For this model, step count was a significant predictor: Each additional 1000 steps showed an increase of 0.206 h in bilateral arm use (B = 0.206, 95% CI [0.172, 0.239], *p* < 0.001). FMA-UE (B = −0.014, 95% CI [−0.047, 0.018], *p* = 0.379) and use of gait aid (B = −0.14, 95% CI [−0.40, 0.12], *p* = 0.285) were not significant predictors of concurrent arm use time during walking.

The regression model predicting unilateral paretic arm use time was statistically significant (F(3, 42) = 6.015, *p* = 0.002); explaining 25.1% of the variance in the outcome after adjustment for model complexity (Adjusted R^2^ = 0.25.1, R^2^ = 0.301). Step count was a significant predictor: Each additional 1000 steps showed an increase of 0.027 h in paretic arm use (B = 0.027, 95% CI [0.011, 0.043], *p* = 0.001). FMA-UE (B = 0.001, 95% CI [−0.014, 0.017], *p* = 0.866) and use of gait aid (B = 0.042, 95% CI [−0.084, 0.168], *p* = 0.502) were not significant predictors of paretic arm use during walking. 

Although the regression model for unilateral non-paretic arm use time was not statistically significant (F(3,42) = 2.050, *p* = 0.121), step count (B = 0.021, 95% CI [−0.001, 0.042], *p* = 0.051) and gait aid use (B = 0.159, 95% CI [−0.009, 0.328], *p* = 0.063) showed non-significant trends toward predicting non-paretic arm use. The regression model for magnitude ratio was not statistically significant (F(3,42) = 1.681, *p* = 0.186) and none of the predictors significantly explained the variance in these outcomes (all *p* > 0.05).

## 4. Discussion

This study explored factors influencing the interpretation of accelerometer-derived measures of real-world UE use in individuals with subacute stroke. The results of this study highlight the importance of characterizing both the context of arm use (i.e., walking vs. non-walking), walking activity (i.e., step count) and mobility aid use when interpreting UE activity in real-world conditions. Bilateral magnitude, a measure of acceleration magnitude across both arms, was influenced by UE activity occurring during continuous walking. Bland–Altman analyses assessing agreement between UE-Total and UE-ADL for bilateral magnitude indicated that participants with higher overall arm activity and greater walking activity (daily step count) exhibited larger between-measure differences. This influence of walking activity was more pronounced when examining agreement between UE-Continuous Walking and UE-ADL, with higher bilateral magnitude during UE-Continuous Walking and larger differences among participants with higher step counts. Mobility aid type was linked to differences in magnitude ratio when looking at agreement between UE-Continuous Walking and UE-ADL, as indicated by the Bland–Altman analysis with proportional bias. Step count was a consistent predictor across UE measures for both UE-Continuous Walking and UE-ADL. Although UE motor impairment (FMA-UE) was a significant predictor of magnitude ratio and unilateral paretic arm use time for UE-ADL, its modest effect size indicates limited explanatory power. Taken together, findings demonstrate that factors such as walking activity and mobility aid use influence IMU-derived metrics of UE use during continuous walking and ADL-based activity and should be carefully considered when making decisions about monitoring UE use in real-world conditions in people with subacute stroke. 

This study assessed the contribution of UE-Continuous Walking on UE-Total while addressing a number of limitations in previous studies. First, some studies did not remove the gravitational component from accelerometer signals [8,18,32], or did not explicitly state whether the raw signal was filtered to remove the effects of gravity prior to calculating the average vector magnitude [11,14,15,24], whereas in the present study gravitational components were removed from the vector magnitude to focus on the dynamic UE use and reduce UE use overestimations that would arise from the contribution of constant acceleration due to gravity. Second, periods of inactivity across both UEs were removed, preventing artificially inflated symmetry when both arms showed no movement. Third, prior studies have used epochs ranging from one second to one minute [8,11,14,15,18,24]. Very short epochs may fragment functional movements into smaller segments that do not reflect meaningful activity or may capture incidental movements that occur due to motion of another body segment rather than goal-directed actions. In contrast, longer epochs may smooth or average distinct movements, potentially hiding brief but functionally relevant UE activity [45]. Thus, we used 5-s epochs to address these issues. Lastly, instead of using device-specific activity counts to quantify UE activity, we calculated our sensor-derived metrics using AVM values. Activity counts are derived from proprietary algorithms that vary by device, limiting reproducibility and cross-study comparability [28]. In contrast, AVM is a raw-signal amplitude-based metric that is transparent and device-agnostic, allowing for consistent calculation across different IMU systems. The current data processing and analysis approach provides a means for moving toward standardized measures, allowing for reproducibility and comparisons across studies. 

In addition to addressing prior limitations, findings from this study provide additional insight to measurements of real-world free-living UE function. Leuenberger et al. (2017) [18] found that arm swing during walking accounts for a large proportion of total arm use time in people post-stroke with severe impairment. In the current study, comparisons between UE-Continuous Walking and UE-ADL revealed important differences, highlighting the need to examine these contexts separately. Results showed that UE activity during continuous walking, reflecting arm swing, is characterized by greater activity across both arms (bilateral magnitude), concurrent use time and increased incorporation of the paretic arm (as indicated by magnitude ratio), resulting in more symmetrical UE use compared with ADL-based activity. This aligns with Bezuidenhout et al., [9] who reported that UE use is more symmetrical during ambulation than during non-ambulatory activities. It has been recommended that rehabilitation promote high dosage and intensity UE practice and incorporation of the paretic UE into daily tasks to support recovery [46]. Separating UE use during walking compared to use during ADLs has implications for the interpretation of real-world use of the paretic UE. For example, individuals who walk more frequently may accumulate substantial UE movement through arm swing despite limited use of the paretic UE during more skilled functional tasks (e.g., dressing, cooking). Distinguishing ambulatory UE activity from ADL-based activity therefore allows clinicians and researchers to determine whether measures of UE activity reflect purposeful engagement of the paretic UE or somewhat passive arm swing during walking. This distinction may provide a more accurate assessment of functional UE use and inform interventions aimed at increasing use of the paretic UE into more skilled functional daily activities.

In line with prior research, our findings further suggest that overall level of activity, such as step count, is an important variable to consider when evaluating sensor-derived measures of UE activity [23,24]. For bilateral magnitude and unilateral paretic use time in both UE-Continuous Walking and UE-ADL, step count accounted for a large portion of the variance, highlighting the importance of considering the level of activity when interpreting UE activity in real-world conditions. In this study, some participants (n = 14) were actively receiving inpatient rehabilitation, with the majority living at home (n = 32). However, many continued to participate in outpatient rehabilitation. Prior research suggests that activity levels may be higher during inpatient rehabilitation [47], where individuals are encouraged to engage in regular therapy and intensive task-specific practice to promote recovery, compared with the home environment where activity is largely self-directed. Consequently, it is possible that differences in UE use patterns between walking and ADL-based activities may be more apparent in inpatient settings, while lower overall activity levels at home may reduce the ability to detect such distinctions. Due to the small sample size and the imbalance in participant representation across the home and inpatient settings, we were unable to assess whether collection setting influenced the relationships between accelerometry-derived UE measures and their predictors. Given these known differences in activity levels between rehabilitation settings, the influence of collection setting on real-world UE use warrants further investigation in future studies. Additionally, the definition for ambulatory and non-ambulatory contexts must be considered carefully when characterizing UE use. For example, Lueunberger et al. [18] alternatively excluded long walking bouts (>7.5 s) and short walking bouts (<7.5 s) to examine differences in UE activity during ambulatory and non-ambulatory periods. As a result of excluding short walking bouts, their non-ambulatory data might not have captured ADLs that involve concurrent UE use together with intermittent stepping, such as performing light household tasks (e.g., cooking, cleaning). In contrast, the algorithm used in this study required consecutive steps to be within 2 s [40], such that steps not meeting these criteria were identified as occurring during ADL-based activities. This allowed for the examination of UE activity during periods characterized by intermittent stepping rather than sustained ambulation. Consistent with this approach, step count was positively associated with higher bilateral magnitude and unilateral paretic arm use time for UE-ADL, indicating that steps taken during non-ambulatory activity were linked to increased use of both arms (e.g., ADLs performed while standing with intermittent stepping activity). These findings align with prior research demonstrating strong associations between ADL performance (e.g., self-care, feeding/cooking) and the ability to ambulate [48]. Taken together, these results suggest that individuals with higher levels of activity as reflected by step count may engage more frequently in activities that involved intermittent stepping and UE use, such as self-care tasks or light housework. 

Although FMA-UE was a significant predictor of unilateral paretic arm use time and magnitude ratio for UE-ADL, the magnitude of change was modest for both. Each 5-point increase in FMA-UE corresponded to a 0.071-h (four minutes per day) increase in paretic arm use and a 0.073-point increase in magnitude ratio. Consistent with prior literature, this highlights a potential disconnect between motor capacity and real-world UE use [8,9,10,22,49]. UE engagement was also influenced by mobility aid use. Subgroup analyses using Bland–Altman plots revealed that magnitude ratio (interlimb symmetry) varied by mobility aid type when comparing UE-Continuous Walking with UE-ADL, with a reversal in slope observed between participants who did not use gait aids and those who used a cane. Overall symmetry played an important role in the observed differences in UE activity; individuals without gait aids demonstrated smaller differences as they became more symmetric (magnitude ratio = 0), whereas cane users exhibited larger differences as they moved closer to symmetry. This pattern suggests that cane users may appear more asymmetric during continuous walking despite exhibiting relatively symmetrical UE use during ADL-based activities, likely reflecting the mechanical and functional demands associated with cane use during ambulation. This complements the findings of Bezuidenhout et al. [9], who reported asymmetric arm use in cane users during walking, which was attributed to the fixed positioning of the non-paretic arm during cane use. Wheeled walker users did not show proportional bias when comparing differences between UE-Continuous Walking and UE-ADL for magnitude ratio, suggesting that differences in interlimb symmetry between the two contexts are similar across the observed range of magnitude ratio for this study. This may be due to relatively uniform interlimb symmetry during continuous walking among wheeled walker users, where arm movement is constrained by the need to hold and operate the walker. Notably, it is possible to see distinct UE use patterns during continuous walking in individuals not using a gait aid, as they may present with notable impairment in the paretic arm. Because recovery of lower limb function may occur more rapidly than recovery of upper limb function [3,4,5], the absence of a gait aid is not aligned with reduced UE impairment. For example, the presence of increased tone and spasticity in the paretic UE is known to result in reduced arm function resulting in reduced arm swing during gait [49], which can introduce asymmetry in UE use during continuous walking. Future studies may benefit from incorporating measures of arm posture to provide additional context for interpreting UE use and engagement for continuous walking and ADL-based activities. 

The findings of this study should be interpreted in the context of certain limitations. To preserve statistical power, cane and wheeled walker users were combined into one group in the regression analyses. While this approach may have reduced sensitivity to aid-specific differences, Bland–Altman analyses suggested distinct patterns in bilateral magnitude and magnitude ratio between cane and wheeled walker users. The study sample included 46 participants across the full range of UE impairment, and the subgroup sizes (mild: n = 29, moderate: n = 9, and severe: n = 8) may have been too small to fully account for interindividual variability. It should be noted that results of this study do not capture changes in UE use over the course of recovery (e.g., subacute to chronic). Consequently, the findings provide a snapshot of real-world UE activity and do not offer insight into recovery trajectories or the longitudinal relationship between real-world UE activity and clinical measures of UE recovery. In addition, there are several individual-level factors that influence real-world use which could not be addressed in this study. For example, 37% of participants had their dominant side affected by stroke, but the sample size did not allow us to explore hand dominance as a covariate. Although there have been inconsistent results regarding the impact of the dominant hand as the paretic limb [23], it remains an important variable to consider, as it can modulate both the degree of impairment’s impact on UE function and use [50,51] and the interpretation of real-world UE use patterns [10]. Lastly, prior research has emphasized the importance of integrating the paretic arm during bilateral UE tasks to promote functional recovery and interlimb coordination [52]. The use of 5-s epochs in the present study may have limited the ability to identify brief (under 5 s) periods of bilateral UE use. 

Considering the complexity of factors affecting UE use, future studies with larger sample sizes are needed to explore the influence of additional contributing variables on UE use in real-world conditions. Future studies should examine whether accelerometry-derived measures of real-world UE use, such as bilateral magnitude and magnitude ratio, are associated with recovery trajectories from the subacute to chronic period of recovery. Although weak to moderate associations have been reported between UE function (Action Research Arm Test scores) and bilateral magnitude and magnitude ratio in chronic stroke [8], no research has established what magnitude of change in these accelerometry-derived measures represents a clinically meaningful improvement. Establishing such benchmarks could enhance interpretation of accelerometry outcomes and support tracking of real-world UE use and recovery as people with stroke progress from the subacute period into the chronic stage.

## 5. Conclusions

The results from this study highlight the complexity of factors that can impact the interpretation of wearable sensor-derived metrics of real-world UE use in subacute stroke. Bilateral magnitude, representing the total magnitude of acceleration across both UEs, was higher for UE-Continuous Walking compared to UE-ADL, highlighting that continuous walking periods show greater arm movement. Although mobility aid use influenced UE use symmetry (magnitude ratio) during continuous walking, considerable variability was present within each mobility aid group. Finally, overall activity level, as indexed by step count, explained a substantial proportion of the variance in UE activity across models during both continuous walking and ADL-based activity. Taken together, these findings highlight the heterogeneous and context-dependent nature of post-stroke UE function and emphasizes the importance of considering walking context, mobility aid use, and ambulatory activity level to better understand UE use and function in real-world conditions for people with subacute stroke.

## Figures and Tables

**Table 1:** Participant Characteristics.

**N**		**46**
Sex, n (%)	Female	18 (39.1)
	Male	28 (60.9)
Age, mean (SD)		61.5 (13.6)
Setting	Hospital	14
	Home	32
Time since stroke (weeks), Mean (SD)		14.4 (4.9)
Type of Stroke, n (%)	Ischemic	37 (80.4)
	Hemorrhagic	9 (19.6)
Mobility Aids, n (%)	Cane	6 (10.9)
	No Gait Aid	21 (45.7)
	Quad Cane	2 (4.3)
	Walking Pole	5 (10.9)
	Wheeled Walker	12 (26.1)
Paretic side, n (%)	Left	30 (65.2)
	Right	16 (34.8)
Dominant hand, n (%)	Left	3 (6.5)
	Right	43 (93.5)
Dominant affected, n (%)	No	29 (63.0)
	Yes	17 (37.0)
**CLINICAL CHARACTERISTICS**		
Fugl–Meyer Upper Extremity (/66), Mean (SD)	48.4 (17.3)	
Fugl–Meyer Lower Extremity (/34), Mean (SD)	29.4 (5.4)	
6 Minute Walk Test (Meters), Median (IQR)	268.5 (131.8–416)	

**Table 2:** Central tendency of IMU-derived daily activity measures across participants.

Step Count per day (continuous walking), Median (IQR)	3740 (2065 to 7739)
Step Count per day (ADL-based), Median (IQR)	877 (698 to 1277)
Total UE Activity: Concurrent use (hrs/day), Mean (SD)	4.4 (1.7)
Total UE Activity: Unilateral Paretic Use (hrs/day), Mean (SD)	1.3 (0.7)
Total UE Activity: Unilateral Non-Paretic Use (hrs/day), Mean (SD)	3.0 (1.6)
Total UE Activity: Sedentary (hrs/day), Mean (SD)	3.2 (1.7)
UE-ADL: Magnitude Ratio, Median (IQR)	−0.9 (−1.3 to −0.5)
UE-ADL: Bilateral Magnitude, Median (IQR)	25.7 (21.7 to 29.4)
UE-Continuous Walking: Magnitude Ratio, Median (IQR)	−0.2 (−0.5 to 0.1)
UE-Continuous Walking: Bilateral Magnitude, Median (IQR)	59.1 (40.6 to 88.1)
Number of Days Worn, Mean (SD)	6.3 (1.5)
Wear time (hrs/day), Mean (SD)	11.9 ± 2.5

**Table 3:** Mean and standard deviation (SD) of time of upper extremity (UE) use shown by condition (UE-Total and UE-ADL) and type of mobility aid used.

	UE-Total			UE-ADL		
	No Gait Aid (n = 21) Mean (SD)	Cane (n = 13) Mean (SD)	Wheeled Walker (n = 12) Mean (SD)	No Gait Aid (n = 21) Mean (SD)	Cane (n = 13) Mean (SD)	Wheeled Walker (n = 12) Mean (SD)
Unilateral: Paretic (hrs/day)	1.5 (0.9)	1.1 (0.5)	1.2 (0.6)	1.3 (0.7)	1.0 (0.5)	1.1 (0.5)
Unilateral: Non-Paretic (hrs/day)	2.7 (1.3)	4.0 (1.9)	3.3 (1.5)	2.6 (1.2)	3.8 (1.7)	3.2 (1.4)
Concurrent (hrs/day)	5.8 (1.5)	4.3 (1.5)	3.6 (1.8)	3.9 (1.1)	3.5 (1.4)	3.1 (1.4)
Sedentary (hrs/day)	3.0 (1.4)	4.1 (2.5)	3.0 (0.9)	2.9 (1.4)	4.0 (2.5)	3.0 (0.9)

**Table 4:** Predictors of UE-ADL. Regression models examined the effects of steps accumulated during ADL-based activities (per 1000 steps), gait aid use (no gait aid vs. gait aid), and Fugl–Meyer Upper Extremity (FMA-UE) score (per 5-point increase) on bilateral magnitude, magnitude ratio, bilateral arm use time, paretic arm use time and non-paretic arm use time. Reported values include unstandardized regression coefficients (B), 95% confidence intervals (CI), t-values, *p*-values, change in R^2^ (ΔR^2^) for each predictor, total R^2^ and adjusted R^2^. Significant *p*-values are bolded in red.

Outcome	Predictor	B	95% CI	T	*p*	ΔR^2^	R^2^	Adjusted R^2^
Bilateral Magnitude	Intercept	27.750	[25.031, 30.469]	20.594	<0.001	—	0.333	0.285
	Steps (per 1000)	4.840	[1.830, 7.850]	3.245	**0.002**	0.134	—	—
	Gait Aid	−3.843	[−7.795, 0.108]	−1.963	0.056 *	0.170	—	—
	FMA-UE (per 5 points)	0.454	[−0.146, 1.055]	1.526	0.134	0.037	—	—
Magnitude Ratio	Constant	−0.781	[−1.070, −0.493]	−5.470	<0.001	—	0.199	0.142
	Steps (per 1000)	0.021	[−0.298, 0.340]	0.135	0.893	0.008	—	—
	Gait Aid	−0.127	[−0.546, 0.292]	−0.612	0.544	0.089	—	—
	FMA-UE (per 5 points)	0.073	[0.009, 0.136]	2.300	**0.026**	0.101	—	—
Concurrent Arm Use (hours)	Constant	3.774	[3.180, 4.368]	12.820	<0.001	—	0.146	0.085
	Steps (per 1000)	0.601	[−0.057, 1.258]	1.844	0.072 *	0.044		
	Gait Aid	−0.332	[−1.195, 0.531]	−0.777	0.442	0.065		
	FMA-UE (per 5 points)	0.091	[−0.040, 0.222]	1.399	0.169	0.040		
Only Paretic Arm Use (hours)	Constant	1.220	[0.953, 1.487]	9.222	<0.001	—	0.216	0.160
	Steps (per 1000)	0.357	[0.062, 0.653]	2.440	**0.019**	0.057		
	Gait Aid	−0.026	[−0.414, 0.362]	−0.136	0.893	0.052		
	FMA-UE (per 5 points)	0.071	[0.012, 0.130]	2.425	**0.020**	0.110		
Only Non-Paretic Arm Use (hours)	Constant	2.629	[1.937, 3.320]	7.674	<0.001	—	0.147	0.086
	Steps (per 1000)	0.546	[−0.219, 1.311]	1.440	0.157	0.053	—	—
	Gait Aid	0.814	[−0.191, 1.818]	1.635	0.109	0.089	—	—
	FMA-UE (per 5 points)	−0.02	[−0.173, 0.133]	−0.261	0.795	0.001	—	—

*p* < 0.05 considered statistically significant; * trends (*p* < 0.10).

**Table 5:** Predictors of UE-Continuous Walking. Regression models examined the effects of steps accumulated during continuous walking (per 1000 steps), gait aid use (no gait aid vs. gait aid), and Fugl–Meyer Upper Extremity (FMA-UE) score (per 5-point increase) on bilateral magnitude, magnitude ratio, bilateral arm use time, paretic arm use time and non-paretic arm use time. Reported values include unstandardized regression coefficients (B), 95% confidence intervals (CI), t-values, *p*-values, change in R^2^ (ΔR^2^) for each predictor, total R^2^ and adjusted R^2^. Significant *p*-values are bolded in red.

Outcome	Predictor	B	95% CI	T	*p*	ΔR^2^	R^2^	Adjusted R^2^
Bilateral Magnitude	Intercept	72.638	[57.124, 88.151]	9.449	<0.001	—	0.398	0.355
	Steps (per 1000)	4.329	[1.337, 7.320]	2.920	**0.006**	0.119	—	—
	Gait Aid	−16.483	[−40.486, 7.521]	−1.386	0.173	0.025	—	—
	FMA-UE (per 5 points)	−0.635	[−3.541, 2.271]	−0.441	0.661	0.003	—	—
Magnitude Ratio	Intercept	−0.307	[−0.740, 0.125]	−1.435	0.159	—	0.107	0.043
	Steps (per 1000)	0.030	[−0.053, 0.113]	0.724	0.473	0.017	—	—
	Gait Aid	−0.131	[−0.800, 0.538]	−0.395	0.695	0.011	—	—
	FMA-UE (per 5 points)	0.039	[−0.042, 0.120]	0.966	0.340	0.020	—	—
Concurrent Arm Use (hours)	Intercept	1.232	[1.059, 1.404]	14.418	<0.001	—	0.882	0.873
	Steps (per 1000)	0.206	[0.172, 0.239]	12.476	**<0.001**	0.441	—	—
	Gait Aid	−0.145	[−0.411, 0.122]	−1.094	0.280	0.002	—	—
	FMA-UE (per 5 points)	−0.014	[−0.047, 0.018]	−0.889	0.379	0.002	—	—
Only Paretic Use (hours)	Intercept	0.078	[−0.004, 0.159]	1.921	0.062	—	0.301	0.251
	Steps (per 1000)	0.027	[0.011, 0.043]	3.471	**0.001**	0.210	—	—
	Gait Aid	0.042	[−0.084, 0.168]	0.677	0.502	0.007	—	—
	FMA-UE (per 5 points)	0.001	[−0.014, 0.017]	0.170	0.866	<0.001	—	—
Only Non-Paretic Use (hours)	Intercept	0.064	[−0.045, 0.173]	1.182	0.244	—	0.128	0.065
	Steps (per 1000)	0.021	[−0.001, 0.042]	2.007	0.051 *	0.075	—	—
	Gait Aid	0.159	[−0.009, 0.328]	1.907	0.063 *	0.109	—	—
	FMA-UE (per 5 points)	−0.008	[−0.029, 0.012]	−0.818	0.418	0.014	—	—

*p* < 0.05 considered statistically significant; * trends (*p* < 0.10).

## Data Availability

The datasets presented in this article are not readily available because the study is currently ongoing. Data will be available upon completion of the study.

## Abbreviations

The following abbreviations are used in this manuscript:

ADL	Activities of Daily Living
AVM	Average Vector Magnitude
CAMAROS	Canadian Maraviroc Randomized Controlled Trial to Augment Rehabilitation Outcomes After Stroke
FMA	Fugl Meyer Assessment
FMA-LE	Fugl Meyer Assessment- Lower Extremity
FMA-UE	Fugl Meyer Assessment- Upper Extremity
IMU	Inertial Measurement Unit
LOA	Limits of Agreement
UE	Upper Extremity
UE-ADL	Upper Extremity- Activities of Daily Living

## Appendix A

**Table A1:** Participant and clinical characteristics by study for the two cohorts pooled for analysis.

		**STUDY 1 (TRANSITION HOME)**	**STUDY 2 (CAMAROS)**	***p*** **-VALUE**
**N**		19	27	N/A
AGE, MEAN (SD)		51.8 (9.9)	67.4 (12.1)	**<0.001**
STROKE TYPE, N (%)	Ischemic	10 (52.6)	27 (100.0)	**<0.001**
SEX, N (%)	Female	8 (42.1)	10 (37.0)	0.968
	Male	11 (57.9)	17 (63.0)	
GAIT AID USE, N (%)	Single Point Cane	1 (5.3)	5 (18.5)	N/A
	No Gait Aid	11 (57.9)	10 (37.0)	
	Quad Cane	1 (5.3)	1 (3.7)	
	Walking Pole	5 (26.3)	0 (0.0)	
	Wheeled Walker	1 (5.3)	11 (40.1)	
TIME SINCE STROKE (WEEKS), MEDIAN (IQR)		14.7 (11.1–20.5)	13.7 (12.4–14.7)	0.294
PARETIC SIDE, N (%)	Left	8 (42.1)	22 (81.5)	**0.014**
	Right	11 (57.9)	5 (18.5)	
DOMINANT SIDE, N (%)	Right	19 (100.0)	24 (88.9)	0.370
DOMINANT AFFECTED, N (%)	No	8 (42.1)	21 (77.8)	**0.031**
	Yes	11 (57.9)	6 (22.2)	
**CLINICAL CHARACTERISTICS**				
FM-UE MEAN (SD)		47.5 (21.3)	46.5 (16.0)	0.787
FM-LE 8, MEAN (SD)		30.2 (5.4)	28.3 (5.7)	0.420
6MWT, MEDIAN (IQR)		416.0 (303–473)	179 (94–334)	**<0.001**
Average Steps, MEDIAN (IQR)		6938 (3364–10618)	2888 (1528–5265)	**0.007**
